# Effects of *Broussonetia papyrifera* (L.) L'Hér. ex Vent. fruits water extract on hippocampal neurogenesis in the treatment of APP/PS1 transgenic mice

**DOI:** 10.3389/fphar.2022.1056614

**Published:** 2022-10-26

**Authors:** Yu-hui Yan, Zi-han Huang, Qing-ping Xiong, Yue-wen Song, Si-yang Li, Bao-wei Yang, Lan Sun, Meng-yuan Zhang, Yu Ji

**Affiliations:** ^1^ School of Pharmacy, Jiangsu Food & Pharmaceutical Science College, Huai’an, Jiangsu, China; ^2^ School of Food Science and Engineering, Jiangsu Ocean University, Lianyungang, Jiangsu, China; ^3^ Jiangsu Key Laboratory of Regional Resource Exploitation and Medicinal Research, Huaiyin Institute of Technology, Huai’an, Jiangsu, China

**Keywords:** *Broussonetia papyrifera* (L.) L'Hér. ex Vent. fruits water extract (BLWE), Alzheiemer’s disease, neurogenesis, metabolomics, wnt/β-catenin signaling pathway

## Abstract

**Background:** Adult neurogenesis plays an important role in repairing damaged neurons and improving cognitive impairment in Alzheimer’s disease (AD). *B. Papyrifera* (L.) L'Hér. ex Vent. fruits (BL), a traditional Chinese medicine for tonifying the kidney, has been reported to improve cognitive function in AD mice, but the underlying mechanisms have not been clearly illuminated. This study aimed to provide an overview of the differential compounds in the brain of APP/PS1 mice after BL water extract (BLWE) treatment through metabolomics technology and to elucidate whether the therapeutic effect and mechanism are through the enhancement of neurogenesis.

**Methods:** APP/PS1 transgenic mice were treated with different doses of BLWE. After 6 weeks of intragastric injection, the therapeutic effects of BLWE on APP/PS1 transgenic mice were determined by the Morris water maze test, immunohistochemistry, hematoxylin & eosin and Nissl staining, enzyme-linked immunosorbent assay and terminal deoxynucleotidyl transferase dUTP nick end labeling staining. Subsequently, metabolomics technology was used to analyze the regulatory effect of BLWE on differential compounds in the brain of APP/PS1 mice, and on this basis, its molecular mechanism of BLWE was screened. Finally, the protein expression of the Wnt/β-catenin signaling pathway was detected by Western blotting.

**Results:** After BLWE treatment, the learning and memory function of APP/PS1 mice were significantly improved, which was related to the increase in the number of Nestin^+^/BrdU^+^ and NeuN^+^/BrdU^+^ cells, and the decrease in the number of apoptotic cells in the hippocampus. BLWE treatment could also up-regulate the expression of synapse-associated proteins. Moreover, BLWE could modulate endogenous metabolic compounds in the brains of AD mice, including N-acetyl-aspartate, glutamine, etc. Furthermore, BLWE inhibited the phosphorylation of Tyr216-GSK-3β and β-catenin protein while increased CyclinD_1_ protein expression.

**Conclusion:** We demonstrated that BLWE can enhance neural stem cells proliferation and improve neurogenesis, thereby efficiently repairing damaged neurons in the hippocampus and ameliorating cognitive impairment in APP/PS1 transgenic mice. The mechanism is at least partly through activating the Wnt/β-catenin signaling pathway.

## Introduction

Alzheimer’s disease (AD) is a common neurodegenerative disorder that affects the elderly. As the population ages, the prevalence of AD is rising, which represents a substantial threat to the health of older people. Among the pathological characteristics of AD is the aberrant amyloid beta peptide (Aβ) deposition in the brain, which may promote neuroinflammatory responses, leading to a high number of synaptic losses and neuronal death in the brain. Consequently, AD patients will lose their capacities for learning and memorization (Golde et al., 2018; Uddin et al., 2020; de Almeida et al., 2022; Shakir and Dugger, 2022). Drugs continue to play a significant role in the treatment of AD, with acetylcholinesterase inhibitors and N-methyl-d-aspartate receptor antagonists being two of the most used types of medications for this condition (Liang et al., 2022). However, these medications may only alleviate the symptoms of certain patients but cannot effectively compensate the loss of neurons in the brain (Schott et al., 2019). Consequently, there is an urgent need for novel treatment strategies.

Neural stem cells (NSCs) are self-renewing cell populations that have the ability to differentiate into neurons, astrocytes, and oligodendrocytes (Jaberi et al., 2021). NSCs transplantation is regarded to be a successful treatment option for AD. However, the inflammatory microenvironment in the AD patients brains is not favorable to the survival of transplanted NSCs, therefore the therapeutic benefit is limited (Llorens-Bobadilla and Martin-Villalba, 2017). It has been hypothesized through previous research that the adult mammalian brain lacks regenerative repair capabilities. The following studies, however, demonstrate that neurogenesis does occur in the adult central nervous system (CNS), allowing for the treatment of CNS degenerative diseases and the repair of CNS injuries (Gage, 2019; Hanspal and Gillotin, 2022). Stimulating the proliferation of endogenous NSCs is considered as a practical way for CNS disease treatment, including AD and other neurodegenerative diseases. Certain traditional Chinese medicinal herbs and their extracts or active compounds have the abilities to modulate the biological characteristics of NSCs and enhance neurogenesis (Chen et al., 2019b). Kong et al. (2015) reported that osthole, an active compound derived from *Angelica biserrata*, can promote neurogenesis in the hippocampus of APP/PS1 transgenic mice. This resulted in an improvement in the mice’s cognitive performance. The active substance Astragaloside VI, derived from *Radix Astrastragali*, promotes the proliferation of NSCs and neurogenesis in a model of transient cerebral ischemia injury, thereby improving the restoration of neurological function in rats with post-ischemic stroke (Chen et al., 2019a).

*Broussonetia papyrifera* (L.) L'Hér. ex Vent. fruits (BL) is a kind of Chinese herbal medicine distributed in most parts of China. Recent studies have revealed that BL extracts have a wide range of pharmacological effects, some of which include boosting the immune system, lowering blood lipids, anti-oxidation, and offering protection against drug-induced liver injury (Pang et al., 2014; Zhang et al., 2020). In addition, recent pharmacological studies have shown that BL extracts have neuroprotective actions on improving cognitive function in AD mouse model and chronic cerebral hypoperfusion rat model (Liu et al., 2020; Li Y. H. et al., 2021). However, it is unknown whether BL water extract (BLWE) can stimulate the growth of endogenous NSCs.

The proliferation, differentiation of NSCs, and neuronal apoptosis are closely related to the Wnt/β-catenin signaling pathway (Yu et al., 2017). Recent studies showed that the expression of glycogen synthase kinase-3β (GSK-3β) and β-catenin, two key molecules of the Wnt/β-catenin signaling pathway, were shown to be significantly different in normal mice and AD model mice (Sun et al., 2019; Menet et al., 2020). However, it is unknown if BLWE could increase the proliferation of endogenous NSCs and whether the Wnt/-catenin signaling pathway is involved in this process. In this study, we aimed to assess the effects of BLWE on the neurogenesis of AD mice and to investigate underlying mechanism.

## Materials and methods

### Animals, drug preparation, and administration

BL was purchased from Kangmei Pharmaceutical Co., Ltd. (Guangdong, China). The voucher specimens were identified by Professor Tian-min Wang, who is working at Liaoning University of Traditional Chinese Medicine, then put in Jiangsu Food & Pharmaceutical Science College for storage (No. JSFPC20191207-01, Huai’an, China). BL (90 g) was soaked in deionized water (900 ml) for 1 h, then heated to boil at 100°C, and maintained in micro-boiling state for 1 h by a thermostat electric heating mantle (DZTW, Shanghai Lichen, China). The extracted solution was collected, and the dregs of a decoction was continued to be extracted twice according the same method, combine the three extracts to obtain the BLWE. After that, the BLWE was concentrated, freeze-dried, and its yielding capacity was calculated by three repeated experiments. The extraction yield of BL was calculated to be 8.48%. 5-Bromo-2-deoxyuridine (BrdU, Sigma Chemical Company, St. Louis, MO, United States) was prepared with deionized to a final concentration of 10 mg/mL and then deposited at 4°C.

APP_swe_/PS1_ΔE9_ transgenic (Tg) mice were purchased from Beijing HFK Bioscience Co., Ltd. (Beijing, China), which can overexpress mutated human APP and PS1 (APP_swe_/PS1_ΔE9_) genes and simulate the pathological features of AD patients. Usually, cognitive impairment can be observed in 7-month-old Tg mice, and Aβ deposits can be detected in the hippocampus and cortex of 10-month-old Tg mice (Zhang et al., 2021). In this study, 9-month-old Tg mice were used and divided into model group, BLWE low dose (l-dose) group, and BLWE high dose (H-dose) group. The dose of mouse was converted according to the dose of human body, that is, 0.15g/kg for BLWE l-dose group and 0.30g/kg for BLWE H-dose group. We treat APP/PS1 mice according to the ‘Guidelines for the Care and Use of Laboratory Animals published by the Shanghai Science and Technology Press of China in 2012. The BLWE groups received different doses of BLWE intragastric treatment once daily for 6 weeks. The model group was given an equal volume of deionized water for 6 weeks, and wild-type C57BL/6 mice at the same age were used as the control group. Three days before the end of the administration, mice were injected with BrdU (50 mg/kg, i. p.) twice a day to label proliferative cells.

### Morris water maze test

Six weeks after administration, the learning and memory abilities of mice were evaluated by the MWM test. The MWM includes a circular pool with a black inner wall, a diameter of 120 cm and a height of 60 cm, a camera, and automatic image acquisition and analysis system. The pool was divided into four quadrants in which a 10 cm diameter platform was placed as an escape platform. Add water (25°C) until the water surface is 2 cm higher than the platform. The swimming paths of mice were captured with the camera, and the images were collected and analysed to assess their spatial acquisition and memory abilities. The operation details could be reviewed from our previous research (Yan et al., 2018).

### Immunohistochemistry

After the MWM experiment, the mice were anesthetized by i. p. with pentobarbital sodium (40 mg/kg), and fixed in the supine position. The brains were taken after cardiac perfusion, placed in the precooled paraformaldehyde solution, and fixed for 4–6 h. A rotary microtome (RM2235, Leica, Germany) was used to prepare paraffin sections (4 μm).

Sections were transparented twice in xylene solution for 15 min each time and then dehydrated by 70%, 80%, and 90% alcohol solution. The sections were placed in 0.01 mol/L sodium citrate buffer (pH = 6.0), heated to boiling in a microwave oven, and the process repeated three times for 5min each time. After permeabilizing the sections with 3% hydrogen peroxide 0.1% Triton X-100 for 30 min, the sections were blocked with 5% bovine serum albumin (BSA) solution for 1 h, then add the primary antibody diluent (rat-anti-Nestin; mouse anti-NeuN; mouse anti-synapsin-1 (SYN-1); mouse anti-Aβ_1-42_, all diluted 200 times, Millipore, Billerica, MA, United States) at 4°C overnight. Then add Cy3-or HRP- labeled secondary antibody diluent (1:250, Jackson ImmunoResearch Lab, West Grove, PA, United States) in the dark for 60 min at room temperature. 4, 6-diamidino-2-phenylindole (DAPI, Sigma-Aldrich, St. Louis, MO, United States) or DAB chromogenic solution was added to each section for 5–15 min, respectively. After washing with PBS, add an anti-fluorescence quencher to mount the slides, observe and take pictures under a fluorescence microscope. As it does in the previous reports (Li H. et al., 2021; Xiao et al., 2021), we use ImageJ software to scan the fluorescence intensity of each field to measure the expression level.

### Enzyme-linked immunosorbent assays

The mouse brain tissue was added to cold normal saline (NS) to prepare 10% homogenates and then centrifuged for 10 min at 3,000 /min. The levels of synaptophysin (SYP), postsynaptic density-95 (PSD-95), interleukin-6 (IL-6), IL-1β and tumor necrosis factor-α (TNF-α) in the supernatant were evaluated by ELISA kit (Wuhan Elite Biotechnology Co., Ltd. Wuhan, China) according to the kit instructions. Add 100 μL of supernatant to a 96-well plate and incubate at 37 C for 90 min 100 μL biotinylated antibody working solution was added immediately after discarding the liquid and then incubated at 37°C for 60 min. Discard the liquid and wash 3 times, then add horseradish peroxidase-conjugated (HRP, Genview, United States) enzyme conjugate working solution 100 μL to each well, incubate at 37°C for 30 min, and then discard the liquid and wash 5 times. Add 90 μL of substrate solution to each well in dark conditions and incubate at 37°C for 15 min. The absorbance of each well at 450 nm was detected using a microplate spectrophotometer immediately after adding 50 μL of stop solution.

### Hematoxylin and eosin and nissl staining

To evaluate the morphological changes of neurons and brain damage in mice, H&E and Nissl staining were performed as previously described (Su et al., 2019; Wang et al., 2019). Briefly, brain sections were dehydrated by 70%, 80%, and 90% alcohol solution and then stained with hematoxylin. Put the slices into 1% hydrochloric acid alcohol solution to fade until the color turns red. After being washed until the color returned blue, the sections incubated with 0.5% ammonia hydroxide and followed by dying with eosin for 5 s, permeabilized with dimethylbenzene after dehydration, and then sealed with neutral gum. For Nissl staining, the brain sections were stained with Cresyl violet after being degreased with different concentrations of alcohol solution (Xiao et al., 2021).

### Terminal deoxynucleotidyl transferase dUTP nick end labeling assay

TUNEL assay can detect apoptotic DNA strand breaks in cell nuclei. The above brain sections were fixed with 4% paraformaldehyde and then permeabilized with 0.1% Triton X-100. After being washed with PBS, they were incubated with PE buffer for 1 h at 37°C and counterstained the nuclear with DAPI. Use an inverted fluorescence microscope to take pictures of brain sections and use ImageJ software to count the TUNEL-positive cells.

### Metabolomics analysis

Using Ultimate 3,000 liquid chromatograph (LC) combined with Q Exactive mass spectrometer (MS, Thermo), we performed metabolomics to investigate the mechanism by which BLWE ameliorates cognitive impairment by promoting nerve regeneration in APP/PS1 mice. A portion of mouse brain tissue is stored at −80°C for 24 h. After thawing, accurately weighed 50 mg and homogenized with 80% cold aqueous methanol 800 μL for 90 s, then vortex and shake to mix well. Ultrasonic extraction at 4°C for 30 min, stand at −20 °C for 1 h, vortex for 30 s, and then stand at 4°C for 0.5 h. After centrifugation (4°C, 12,000 rpm, 15 min), transfer all supernatant to a new centrifuge tube and let them stand at −20°C for 1 h, then centrifuged again under the above conditions. Pipette 200 μL of the supernatant, add 5 μL of internal standard dichlorophenylalanine (140 μg/mL) and transfer to a sample vial for LC-MS analysis.

The chromatographic column for LC-MS is ACQUITY UPLC HSS T3 column (100 mm × 2.1 mm × 1.8 μm) and was carried out in positive ion mode. The column temperature was 40°C, and a flow rate of 0.3 ml/min was used in this study. 0.05% formic acid aqueous solution and acetonitrile solution were used as mobile phase A and mobile phase B, respectively. Inject 4 μL sample at the injection temperature of 4°C for determination. The heater temperature of MS was 300°C, following 45 arb sheath gas flow rate, 15 arb auxiliary gas flow rate, and 1arb exhaust gas flow rate. The electrospray voltage and capillary temperatures were 3.0 KV and 350°C, respectively.

The original data underwent peak alignment, retention time correction, and peak area extraction. The identification of the metabolite structure adopts accurate mass matching (<30 ppm) and secondary spectrum matching and searches the Human Metabolome Database (HMDB). Then use autoscaling or UV method to normalize the data. Use MetaboAnalysis tool suite, SIMCA-P software (Umetrics AB, Umea, Sweden), and Kyoto Encyclopedia of Genes and Genomes (KEGG) databases for multi-dimensional statistical analysis and single-dimensional statistical analysis, including principal component analysis (PCA), partial least squares discriminant analysis (PLS-DA), Students’ t-test, multiple variation analysis, and pathway enrichment analysis (Ji et al., 2018).

### Western blot analysis

WB analysis was performed according to the method in the literature (Yan et al., 2017; Xiao et al., 2022). Briefly, after the brain tissue of each mouse was weighed, Lysis Buffer, a protease inhibitor, a phosphatase inhibitor, and PMSF were added in sequence according to the manufacturer’s instructions (R&D, Emeryville, CA, United States) to extract the total protein. 8% SDS-PAGE was formulated to fractionate the protein and then transferred it onto PVDF membranes. After transfer, the PVDF membrane was washed with TBST and then blocked with 5% BSA. The membrane was probed with primary antibodies for 18 h at 4°C as follows: rabbit polyclonal antibody against t-GSK-3β, t-β-catenin, p-β-catenin (1:1000, Cell signaling, United States), mouse polyclonal antibody against p-Tyr216-GSK-3β, CyclinD_1_ and GAPDH (1:1000, Santa Cruz, United States). After washing three times, the membrane was put into HRP-labeled anti-rabbit or anti-mouse secondary antibodies and incubated at room temperature for 1 h. The proteins were visualized with Electrochemiluminescence (ECL) WB detection reagents (Millipore, Billerica, MA, United States) and then analyzed using ImageJ software.

### Statistical analysis

All experiments were repeated at least 3 times, and data were expressed as means ± SD. The differences among multiple groups were analysed by one-way analysis of variance (ANOVA) followed by Tukey’s post-hoc test. The statistical significance between the two groups was analysed by two-way ANOVA followed by Bonferroni post-hoc test. The above analysis was performed using the SPSS version13.0 statistics software, and *p* < 0.05 was considered statistically significant.

## Results

### BLWE improved cognitive impairment in APP/PS1 mice

**FIGURE 1:**
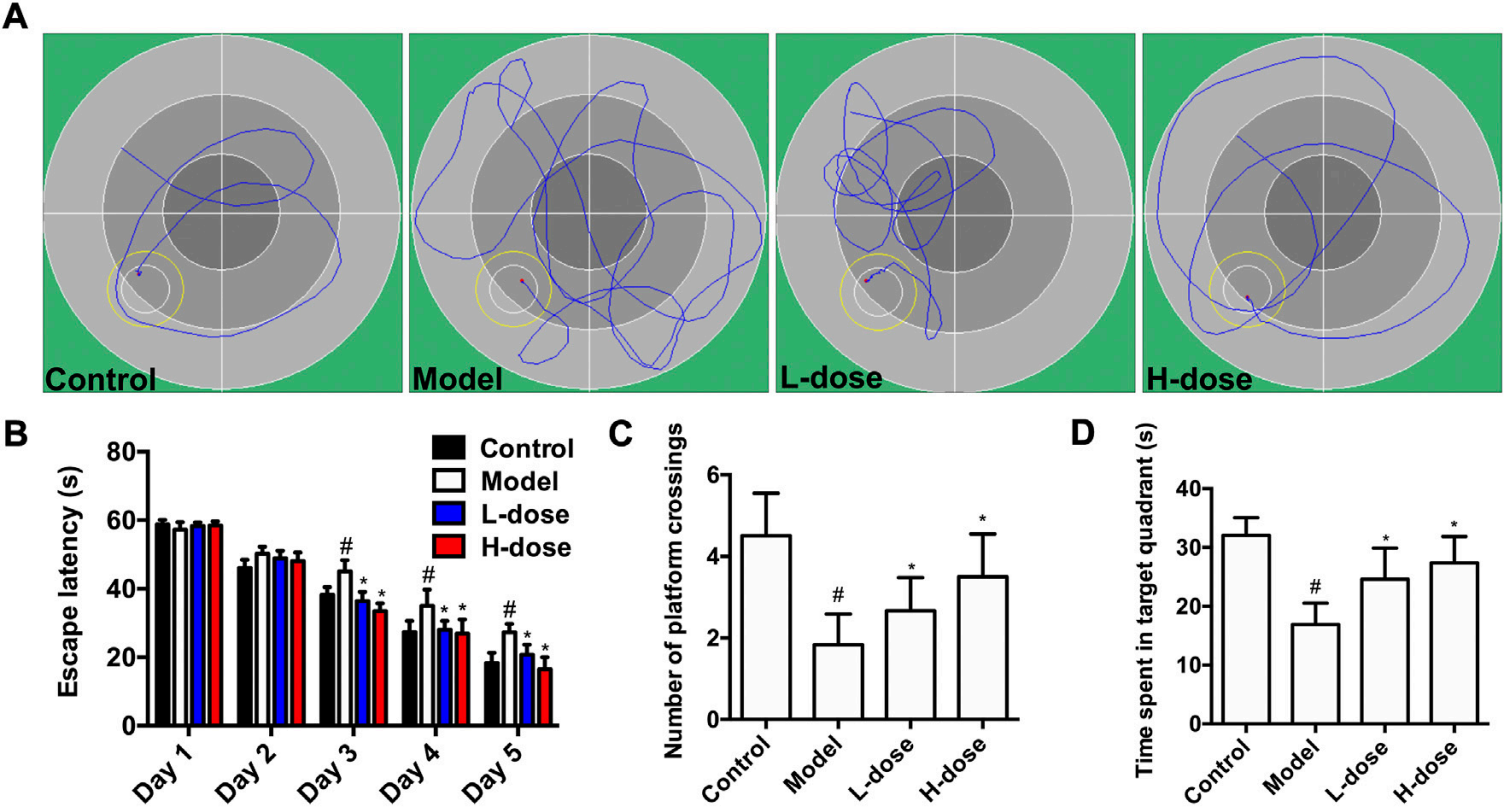
BLWE improved learning and memory ability in APP/PS1 mice. Representative individual swimming trajectory diagram in the water maze trial of each group (at day 5) **(A)**. Comparison of escape latency in different groups of mice in place navigation test **(B)**. Comparison of the number of platform crossings in each group in the space exploration experiment **(C)**. Comparison of the time spent in target quadrant of each group in the space exploration experiment **(D)**. ^#^ *p* < 0.05 vs. the control group; ^*^ *p* < 0.05 vs. the model group. Values were expressed as the mean ± SD (*n* = 6 per group).

A water maze test was first performed to explore the improvement effect of BLWE on the learning and memory abilities of AD mice. The representative swimming trajectory diagram of each group is shown in Figure 1A. The place navigation test results showed that, with the extension of training time, the escape latency of mice in each group gradually shortened, and there were clearly differences among each group after 4 days of training. At day 5, the mice in the control group had the shortest escape latency at 18.32 s, while those in the model group had the longest escape latency at 27.33 s, and there was a significant difference between the two groups (*p* < 0.05, Figure 1B). Meanwhile, after the mice were treated with low and high doses of BLWE, their escape latency was significantly shortened to 20.85 s and 16.57 s compared with the model group, respectively (*p* < 0.05, Figure 1B).

Subsequently, the spatial probe test was performed to detect the maintenance of memory function. As shown in Figure 1C, the average number of platform crossings in the model group was 1.83, significantly different from that in the control group (*p* < 0.05). However, the average number of platform crossings was increased considerably after BLWE treatment (2.67 in the BLWE l-dose group and 3.50 in the BLWE H-dose group); both were statistically significant compared with the model group (*p* < 0.05). In addition, the swimming times of mice in the target quadrant in the BLWE group were significantly prolonged than that in the model group (16.90 s in the model group vs. 24.62 s in the BLWE l-dose group and 27.40 s in the BLWE H-dose group, *p* < 0.05, Figure 1D). The above results indicated that BLWE treatment could enhance both the learning and memory function of APP/PS1 mice.

### BLWE attenuated the deposition of Aβ and inflammatory response in APP/PS1 mice

**FIGURE 2:**
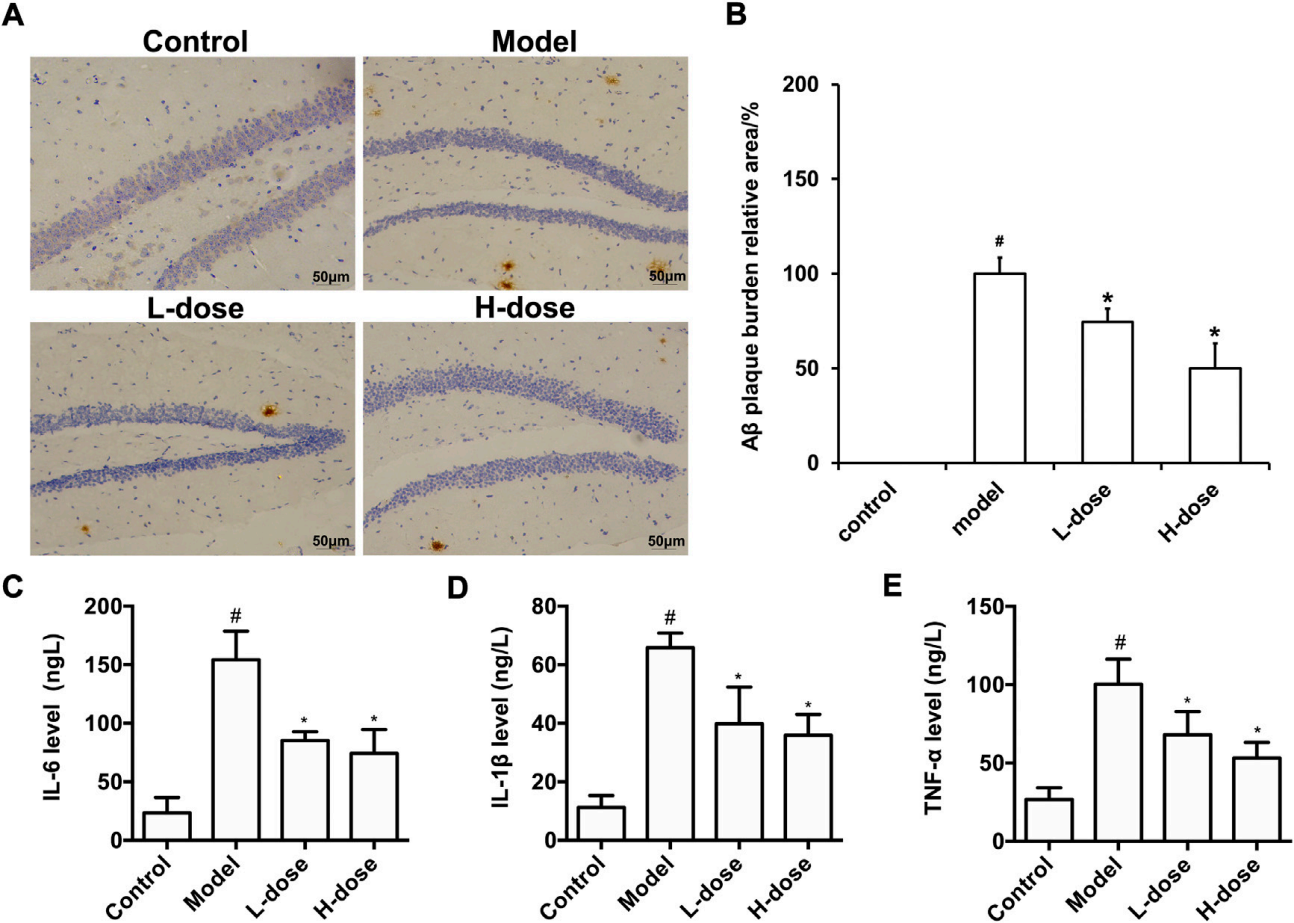
BLWE attenuated the deposition of Aβ and inflammatory response in APP/PS1 mice. Aβ immunoreactive plaques in the hippocampus was immunostained with anti-Aβ monoclonal antibody **(A)**, Scale bar = 50 μm. ImageJ analyzed the percentage area of Aβ-immunoreactive plaques of each group **(B)**. Detection of IL-6 **(C)**, IL-1β **(D)** and TNF-α **(E)** levels in brain tissue of mice in each group by ELISA kit. ^#^ *p* < 0.05 vs. the control group; ^*^ *p* < 0.05 vs. the model group. Values are expressed as the mean ± SD (*n* = 6 per group).

After the MWM test, immunohistochemistry stained the Aβ burden in the brains of mice in each group. The results revealed that there was no Aβ plaque deposition in the control group, while obvious brownish-yellow deposition of Aβ plaques of different sizes and diffusely distributed could be observed in the brain of the model group (Figure 2A). Compared with the model group, BLWE treatment significantly reduced the Aβ plaque positive area in the brain, which was the most significant reduction in the BLWE H-dose group (Figure 2B, *p* < 0.05).

The abnormal deposition of Aβ can activate microglia in the brain, thus promoting the release of inflammatory factors, such as TNF- α, IL-1β, and IL-6. The released inflammatory factors will cause inflammation response in the brain, accelerate the formation of senile plaques due to nerve fiber tangles, and further worsen the course of AD. In order to evaluate the effect of BLWE on the inflammatory reaction in the brain of AD mice, the levels of IL-6, IL-1β, and TNF-α in the whole brain tissue were analyzed by an ELISA kit. The results exhibited that the levels of IL-6, IL-1β, and TNF-α were increased significantly in the brain of the model group, and after BLWE treatment, the levels of these inflammatory factors were significantly reduced, indicating that BLWE can inhibit the inflammatory reaction in the brain (Figures 2C–E, *p* < 0.05).

### BLWE promoted endogenous NSCs proliferation and increased the number of newborn neurons in APP/PS1 mice

**FIGURE 3:**
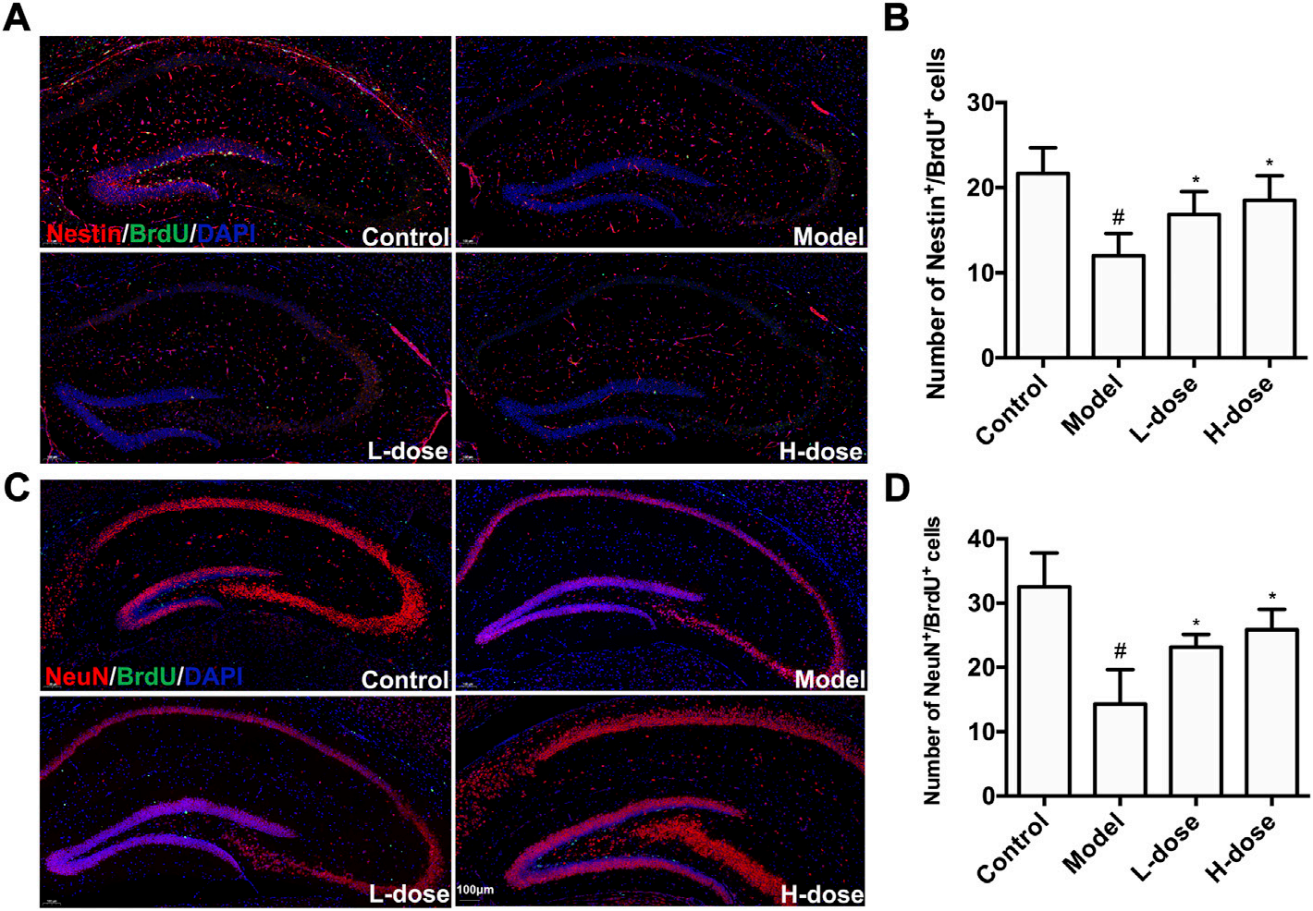
BLWE enhanced endogenous NSCs proliferation and differentiates into neurons in APP/PS1 mice. Mice were treated with different dose of BLWE or deionized water for 6 weeks and were injected with BrdU twice a day to label proliferative cells. Proliferating NSCs were labeled with antibodies against Nestin (red) and BrdU (green), respectively **(A)**. ImageJ software counted Nestin/BrdU positive cells in each group **(B)**. Newborn neurons were labeled with antibodies against NeuN (red) and BrdU (green), respectively **(C)**. ImageJ software counted NeuN/BrdU positive cells in each group **(D)**. The nucleus is counterstained with DAPI (blue). Scale bar = 100 μm in A and C. ^#^ *p* < 0.05 vs. the control group; ^*^ *p* < 0.05 vs. the model group. Values are expressed as the mean ± SD (*n* = 6 per group).

Proliferating NSCs were labeled with Nestin^+^/BrdU^+^ to test and verify the effect of BLWE on neurogenesis. As shown in Figure 3A,B, the Nestin^+^/BrdU^+^ cells in the model group were only 12.00 ± 2.61, which was significantly reduced compared with 21.67 ± 3.01 in the control group (*p* < 0.05). At the same time, we observed that both BLWE l-dose and H-dose treatment can significantly increase the number of Nestin^+^/BrdU^+^ cells in the brains of AD mice (16.83 ± 2.71 cells in the l-dose group and 18.50 ± 2.88 cells in the H-dose group vs. the model group, *p* < 0.05). These results indicate that BLWE can enhance the proliferation of endogenous NSCs in APP/PS1 mouse brains.

Next, we further explored whether BLWE treatment can increase the number of newborn neurons in APP/PS1 mice. Immunostaining against NeuN was used to label neurons, and BrdU was used to label proliferative cells. It can be observed in Figure 3C,D significantly fewer NeuN^+^/BrdU^+^ cells were detected in the model group (14.33 ± 5.32 cells) than that in the control group (32.50 ± 5.22, *p* < 0.05). In contrast, in both BLWE l-dose and BLWE H-dose groups, the number of NeuN^+^/BrdU^+^ cells increased significantly (14.33 ± 5.32 cells in the model group vs. 23.17 ± 1.94 cells in the BLWE l-dose group and 25.83 ± 3.19 cells in the BLWE H-dose group, *p* < 0.05). These findings indicated that BLWE treatments promote the proliferating NSCs differentiating into new neurons in the brain of APP/PS1 mice.

### BLWE increased the expression of synapse-associated protein in APP/PS1 mice

**FIGURE 4:**
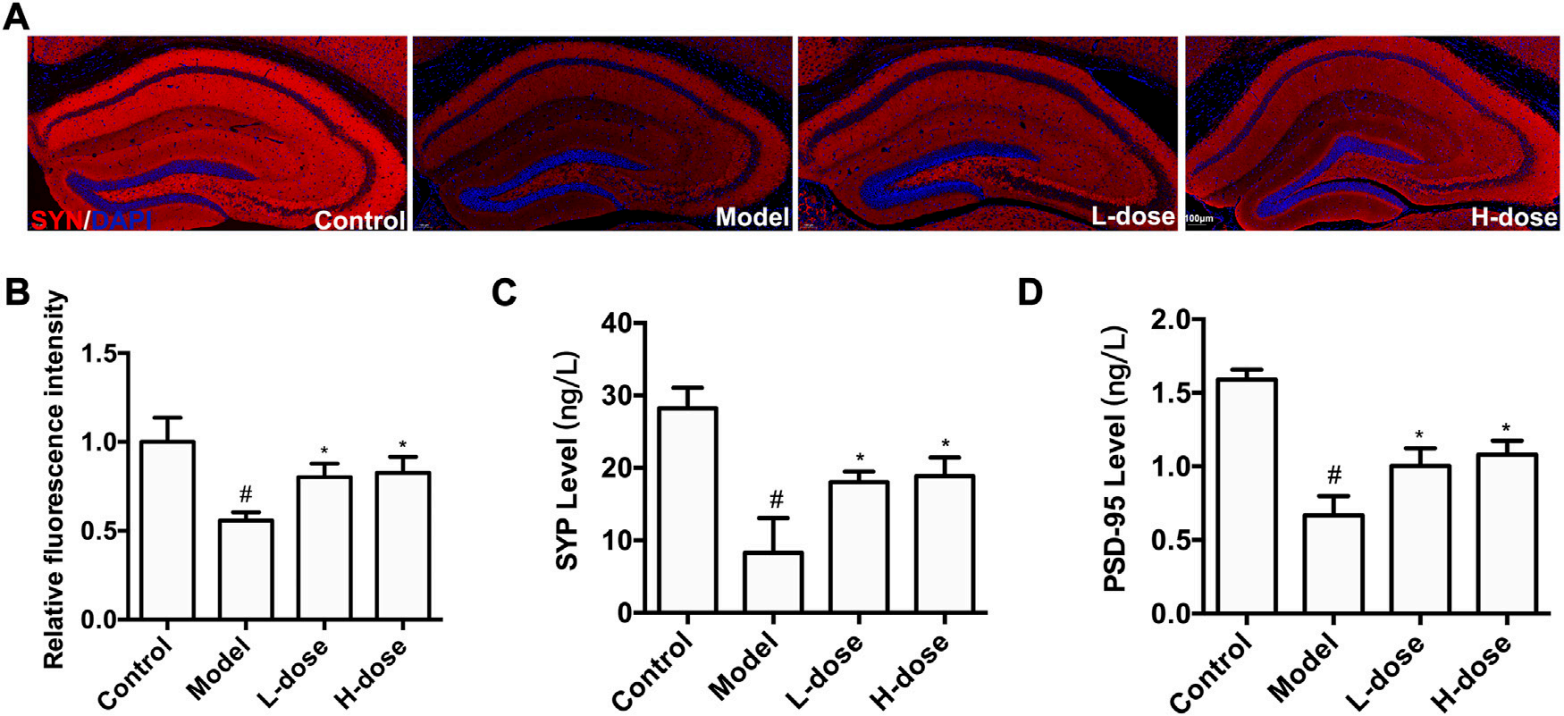
BLWE up-regulated the expression of synapse-associated proteins in APP/PS1 mice. Immunocytochemistry staining for SYN-1 (red) and DAPI (blue) in the brain of each group **(A)**, Scale bar = 100 μm. Quantification of the SYN-1 immunofluorescence intensity by ImageJ software **(B)**. Quantification the concentration of SYP **(C)** and PSD-95 **(D)** by ELISA kit. ^#^ *p* < 0.05 vs. the control group; ^*^ *p* < 0.05 vs. the model group. Values are expressed as the mean ± SD (*n* = 6 per group).

Furthermore, to determine whether new neurons can establish synaptic connections, we used immunostaining and ELISA methods to detect three important protein markers, including SYN-1, SYP, and PSD-95, which were associated with regulating synaptic plasticity and synaptic transmission (Luo et al., 2019; Muhammad et al., 2019). The fluorescence of SYN-1 in each group was shown in Figure 4A, and the expression was quantitatively analyzed using ImageJ software. Compared with the control group, the SYN-1 protein expression was significantly reduced in the model group (*p* < 0.05, Figure 4B). Nonetheless, BLWE therapy effectively restored the expression of the SYN-1 protein (*p* < 0.05 vs. the model group, Figure 4B).

Subsequently, ELISA method was used to detect the level of PSD-95 and SYP. Results in our study demonstrated that compared with the control group, these two proteins significantly decreased in the model group (*p* < 0.05, Figure 4C,D), which could be reversed significantly by BLWE treatment (*p* < 0.05, vs. the model group, Figure 4C,D). These results indicate that BLWE can improve the expression of synapse-related proteins, which is beneficial to the repair of nerve function.

### BLWE inhibited neuron pathological damage and neuronal apoptosis in APP/PS1 mice

**FIGURE 5:**
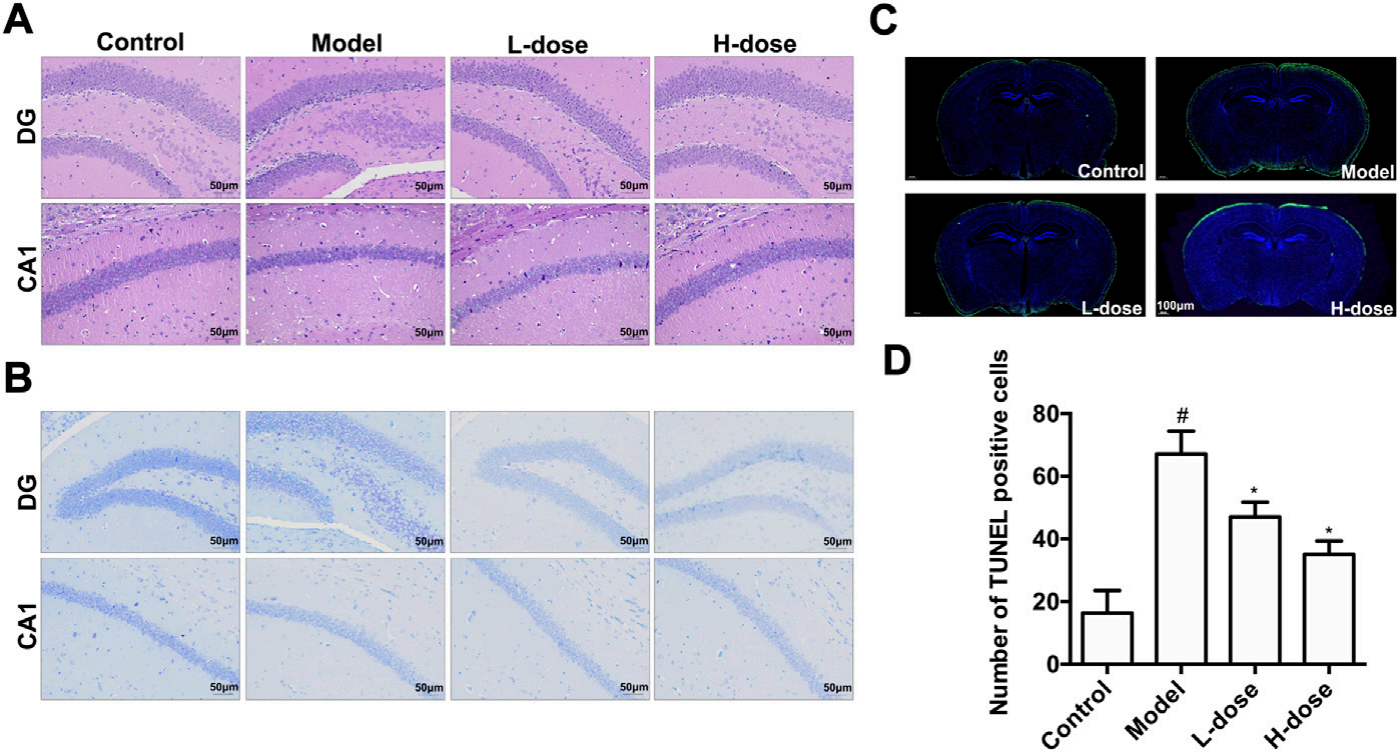
BLWE reversed neuron pathological damage and neuronal apoptosis in APP/PS1 mice. H&E staining of hippocampus DG and CA1 areas **(A)**, Scale bar = 50 μm. Nissl staining of hippocampus DG and CA1 areas **(B)**, Scale bar = 50 μm. TUNEL staining of hippocampus DG and CA1 areas **(C)**, Scale bar = 100 μm. ImageJ software for quantitative counting of apoptotic cells **(D)**. ^#^ *p* < 0.05 vs. the control group; ^*^ *p* < 0.05 vs. the model group. Values are expressed as the mean ± SD (*n* = 6 per group).

We used H&E, Nissl staining, and TUNEL assays to detect neuron pathological damage and apoptosis. In the control group, H&E staining revealed no significant neuronal pathological damage in the hippocampus, DG, and CA1 regions. In contrast, the model group has shrunken neurons, condensed nuclei, and blood cell congestion. Even while neuronal pathological damage was also observed in the two BLWE-treated groups, it was less severe than in the model group (Figure 5A). Nissl staining was subsequently conducted to elucidate the extent of neuron loss in the brain of APP/PS1 mice. The results indicated that in the control group, the hippocampus, DG, and CA1 area cells were neatly arranged, and the Nissl bodies were tigroid or dotted, while the Nissl bodies in the model group were blurred and the cell bodies were shrunken. After treatment with different doses of BLWE, a significant improvement in cell morphology can be observed (Figure 5B).

Moreover, we utilized the TUNEL assay to explore the improvement of neuronal apoptosis by BLWE. As shown in Figure 5C, the green fluorescent staining is TUNEL positive cells. Using ImageJ quantitative analysis, it was shown that the number of TUNEL-positive cells in the control group was only 16.33 ± 2.20 cells, while it was significantly increased to 67.17 ± 7.25 cells in the model group (*p* < 0.05, Figure 5D). Nevertheless, the number of TUNEL-positive cells was decreased in the two BLWE groups (47.01 ± 4.77 cells in the BLWE l-dose group and 35.08 ± 4.34 cells in the BLWE H-dose group), which were significantly different from the model group (*p* < 0.05, Figure 5D), indicating that BLWE could extenuate neuronal apoptosis in APP/PS1 mice.

### Metabolomics analysis of BLWE in AD treatment

**FIGURE 6:**
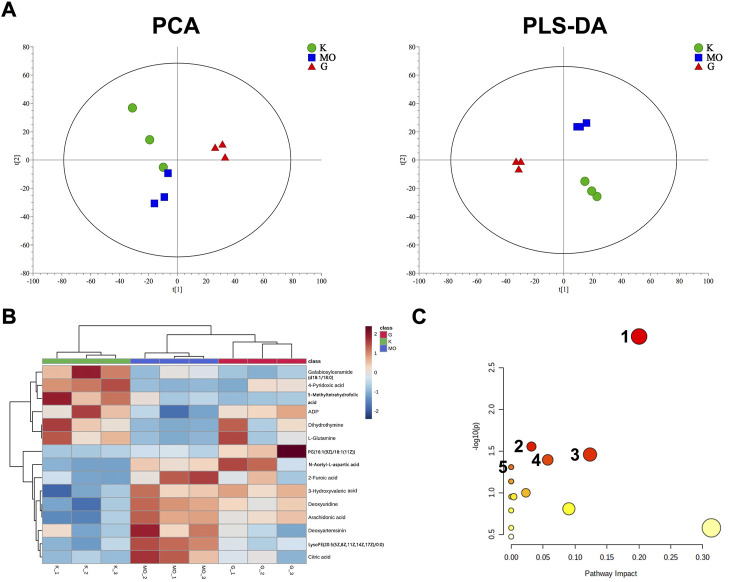
BLWE regulated different metabolites in the brain of APP/PS1 mice. Analysis of PCA and PLS-DA in brain tissue of mice. K denotes the control group; MO denotes the model group and G denotes the BLWE H-dose group **(A)**. Thermal map analysis of differential biomarkers. Metabolites marked in red represent up-regulated metabolites, while those marked in blue represent down-regulated metabolites. Differential metabolites were displayed in each row, and single samples were listed in each column **(B)**. *n* = 3 per group. Overview of BLWE-regulated metabolic pathways. 1, 2, 3, 4, and 5 refer to the alanine, aspartate and glutamate metabolism pathway, glyoxylate, and dicarboxylate metabolism pathway, glycerophospholipid metabolism pathway, pyrimidine metabolism pathway and d-glutamine and d-glutamate metabolism pathway, respectively **(C)**.

Metabolomics analysis was performed to illustrate the mechanism of anti-AD of BLWE. The results of PCA and PLS-DA analysis showed that the samples of each group have high aggregation, and the three groups K (control group), MO (model group), and G (the BLWE H-dose group), can be distinguished by the PLS-DA analysis (Figure 6A). The control group, model group, and BLWE H-dose group can be basically divided, indicating that there are certain differences in metabolism among the three groups. As shown in Table 1, 13 different compounds were screened by KEGG database. Subsequently, the heat map results showed that the metabolites in the brain tissue of mice had significant changes after BLWE treatment. Compared with the model group, eight metabolites, including N-acetyl-aspartate (NAA), glutamine, lactoseceramide, 4-pyridoxic acid, 5-methyltetrahydrofolic acid, ADP, dihydrothymine, and phosphatidylglycerol were increased, and five metabolites including citric acid, 2-furoic acid, deoxyuridine, arachidonic acid, and phosphatidylethanolamine were decreased (Figure 6B). Using the MetaboAnalyst 3.0 software, we conducted an in-depth analysis of the 13 differentially identified compounds to screen out the compounds that are related to the pathogenesis of AD and that BLWE can regulate. Results showed that there were 14 metabolic pathways that BLWE might regulate. Sorted according to the degree of correlation, the top five influences were Alanine, aspartate and glutamate metabolism, Glyoxylate and dicarboxylate metabolism, Glycerophospholipid metabolism, Pyrimidine metabolism, d-glutamine and d-glutamate metabolism (Figure 6C).

**TABLE 1:** Differential metabolites screen by KEGG.

	M1.VIP [5]	M/Z	HMDB	Name	KEGG ID
1	1.54186	192.02678	HMDB0000094	Citric acid	C00158
2	1.4793	174.0396	HMDB0000812	N-Acetyl-l-aspartic acid	C01042
3	1.47922	182.0449	HMDB0000017	4-Pyridoxic acid	C00847
4	1.30564	745.50262	HMDB0010574	Phosphatidylglycerol	C00344
5	1.44032	303.2327	HMDB0001043	Arachidonic acid	C00219
6	1.44016	227.0665	HMDB0000012	Deoxyuridine	C00526
7	1.39829	145.0606	HMDB0000641	l-Glutamine	C00064
8	1.35857	127.05	HMDB0000079	Dihydrothymine	C00906
9	1.34534	458.1668	HMDB0001396	5-Methyltetrahydrofolic acid	C00440
10	1.32724	888.6236	HMDB0004866	lactosylceramide	C01290
11	1.46154	498.26245	HMDB0005779	Phosphatidylethanolamine	C00350
12	1.29828	111.0074	HMDB0000617	2-Furoic acid	C01546
13	1.2915	426.0218	HMDB0001341	ADP	C00008

To further screen the most likely intervening signaling pathways of BLWE, we imported the differential metabolites into the KEGG database for query, matching, and finally found that the affected signaling pathways included Wnt, MAPK, RAS, etc. The Wnt signaling pathway might be the most relevant pathway for BLWE treatment of AD, as most differential biomarkers were associated with it.

### BLWE enhanced neurogenesis in a Wnt signaling-dependent way

**FIGURE 7:**
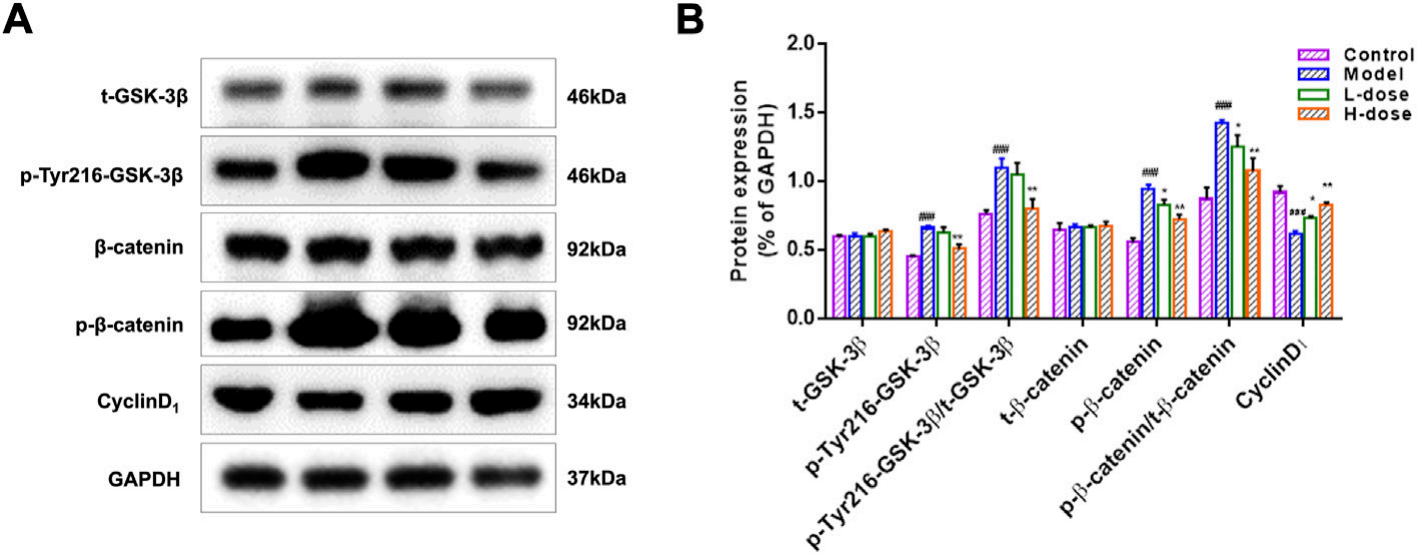
BLWE activated the expression of Wnt/β-catenin signaling pathway-associated proteins in APP/PS1 mice. Protein expression of t-GSK-3β, p-Tyr216-GSK-3β, t-β-catenin, and p-β-catenin in the mice brain measured by WB, GAPDH served as control **(A)**. ImageJ software to quantify protein expression and normalized with GAPDH internal control **(B)**. ^###^ *p* < 0.001 vs. the control group; ^*^ *p* < 0.05, ^**^ *p* < 0.01 vs. the model group. Values are expressed as the mean ± SD (*n* = 6 per group).

It is generally accepted that the activation of the Wnt/β-catenin signaling pathway affects neurogenesis (Kriska et al., 2021). To further investigate the mechanism of BLWE promoting neurogenesis in the treatment of AD, we analyzed the expression of proteins such as t-GSK-3β, p-Tyr216-GSK-3β, t-β-catenin, p-β-catenin, and CylinD_1_, which were related to the Wnt/β-catenin signaling pathway (Figure 7A,B). Results showed that compared with the control group, the expression of p-Tyr216-GSK-3β, p-Tyr216-GSK-3β/t-GSK-3β, p-β-catenin, and p-β-catenin/t-β-catenin were markedly increased, while the expression of CyclinD_1_ was decreased in the model group (*p* < 0.001, Figure 7B). However, the expression of these above proteins can be reversed by BLWE treatment in which the levels of p-Tyr216-GSK-3β, p-Tyr216-GSK-3β/t-GSK-3β, p-β-catenin, and p-β-catenin/t-β-catenin were all down-regulated except the CyclinD_1_ level that was up-regulated significantly (*p* < 0.01, *p* < 0.05, Figure 7B). In addition, there was no significant difference in the protein expression of t-GSK-3β and t-β-catenin among all groups. From the above results, we could conclude that BLWE enhanced neurogenesis through the Wnt/β-catenin signaling pathway.

## Discussion

Combined with pharmacological and metabolomics technology, this study examined the effects and molecular mechanism of BLWE in promoting endogenous NSCs proliferation and regenerative repair for AD treatment. Results confirmed that BLWE treatment for 6 weeks could activate endogenous NSCs proliferation, promote their differentiation into neurons, and establish synaptic networks, as well as reduce neuron pathological damage and neuronal apoptosis in the hippocampus, thereby enhancing the cognitive function of AD mice.

AD is a complex neurodegenerative disease characterized by Aβ deposits, high tau protein phosphorylation, and neurofibrillary tangles in the cerebral cortex and hippocampus. The persistent inflammation and oxidative stress damage around the brain lesion lead to a large number of neuronal ulcerations and losses. The patient exhibited memory loss, cognitive and behavioral disorders, and eventually lost self-care abilities. At present, therapeutic drugs for AD can only compensate for the neurotransmitter that is lacking due to the loss of a large number of neurons but cannot directly supplement the neuron. Therefore, they can only partially alleviate the symptoms of mild and moderate AD patients and have serious side effects, which cannot fundamentally control the natural course of AD (Schott et al., 2019). Most mammals, including humans, have NSCs survive in the subventricular zone (SVZ) and the subgranular zone (SGZ) throughout their lives. After proliferation, the NSCs in the SGZ migrate to the granular layer of the hippocampus, and ulnar gyrus, differentiate into mature neurons and integrate their functions into the neural circuit, thereby making up for the missing neurons in the lesion. This is essential for maintaining the normal function of the hippocampus, such as the learning and memory functions (Gage, 2019; Ou et al., 2022).

We first performed MWM tests to determine the cognitive function in mice. The results indicated that the 6-week treatment of BLWE could shorten the escape latency, increase the number of plate crossings, and prolong the swimming time in the target quadrant to enhance the cognitive function of AD mice (Figures 1A–D). Secondly, we detected Aβ_1-42_ burden to explore whether BLWE can improve the typical pathological features of AD mice and found that Aβ_1-42_ deposition in the brains of mice treated with BLWE was significantly reduced (Figure 2). Further, we separately detected the number of Nestin^+^/BrdU^+^ and NeuN^+^/BrdU^+^ cells, the expression of synapse-related proteins to determine whether neurogenesis increases after treating with BLWE. Results show that, compared to the model group, the number of Nestin^+^/BrdU^+^ and NeuN^+^/BrdU^+^ cells increased significantly in the BLWE group (Figures 3A–D), indicating that BLWE could facilitate the proliferation of endogenous NSCs in the brains of AD mice and promote them to differentiate into neurons. In the process of neuron development, the formation of synaptic connections is the basis for the function of the CNS and a key step for the development of memory. SYN-1 and SYP are specific proteins located in the presynaptic membrane, which are mainly responsible for regulating the release of neurotransmitters in synaptic vesicles, thereby participating in the transmission of chemical information. PSD-95 is the main scaffold protein located in the post-protruding membrane and plays an important role in the synaptic structure and functional plasticity, and then participates in the stability and development of the synapse (Liu et al., 2018; Royero et al., 2020; Yang et al., 2022). Our results also showed the levels of SYN-1, SYP, and PSD-95 in the model group were down regulated, while BLWE could up-regulate the level of the above synaptic proteins (Figure 4). It demonstrated that BLWE could improve synaptic plasticity, which was one of the reasons why it enhanced the cognitive function of AD mice. More importantly, through the results of H&E, Nissl, and TUNEL staining, we found that BLWE can significantly reduce the neuropathological damage caused by AD, as well as reduce neuronal cell necrosis and apoptosis (Figures 5A–D). In summary, the above experimental results were strong evidence to prove that BLWE promotes neurogenesis, thereby treating AD.

Using metabolomics technology to identify differential metabolites and analyze the metabolic pathways has important guiding significance for exploring the drug’s mechanism. Gong et al. (2015) found and identified 19 biomarkers in AD model mice induced by intracerebral injection of Aβ_1-42_ by UHPLC-TOF/MS method, including proline, valine, tryptophan, LPC, plant sphingosine, and fatty acids, etc. Some scholars found that the metabolism of NAA, choline and inositol in the brain tissue of AD patients differs from that of healthy patients, and the results have certain specificity (Glodzik et al., 2015; Lin et al., 2018; Kirov et al., 2021). In our study, we used the LC-MS method to analyze the brain tissue of mice and found 13 metabolites were changed significantly, including NAA, glutamine, lactoseceramide, 4-pyridoxic acid, 5-methyltetrahydrofolic acid, ADP, dihydrothymine, and phosphatidylglycerol, etc. NAA is mainly concentrated in neurons, and its decrease usually represents neurons’ loss, injury, and functional defects. Most studies revealed an increase in NAA in the treatment of AD with cholinesterase inhibitors (Paslakis et al., 2014). Glutamine is the most abundant amino acid in cerebrospinal fluid and is essential for a variety of central nervous system processes, such as depression, pain, and cognitive function. Studies indicate that a drastically decreased glutamine level in the brain may be one of the diagnostic criteria for early AD patients. (Huang et al., 2017). Our research also revealed that the changes of these two important metabolites in the brains of AD mice are similar to those of AD patients. More importantly, these two metabolites can be significantly up-regulated after treatment with BLWE, which may be the main reason for improving neurological function.

By analyzing different metabolites, we determined the metabolic pathways that BLWE might affect, and then used the KEGG database to identify the signaling pathways that may also be influenced. Our results show that the Wnt signaling pathway is the most relevant to the effect of BLWE in the treatment of AD patients. The canonical Wnt signaling pathway was considered closely related to the occurrence, development, and deterioration of neurodegenerative diseases such as AD and PD (Tapia-Rojas and Inestrosa, 2018; Wu et al., 2018). Notably, neurogenesis in the hippocampus was significantly reduced when Wnt signaling was blocked (Casse et al., 2018). In the Wnt/β-catenin pathway, β-catenin is an important cytoplasmic central effector, and GSK-3β is an important negative regulator which could participate in the phosphorylation of β-catenin. Tyr216 is the excitatory phosphorylation site of GSK-3β protein. When the expression of p-Tyr216-GSK-3β decreases, the activity of GSK-3β is inhibited, which can inhibit the phosphorylation of β-catenin, hence increasing the activity of β-catenin, augment the transduction of Wnt signal and then initiate the transcription of downstream CyclinD_1_ (Zeng et al., 2019). CyclinD_1_ is one of the important factors downstream of the Wnt/β-catenin signaling pathway, which is related to the process of neurodegenerative diseases such as AD and PD. Researches have shown that activating the Wnt/β-catenin signaling pathway can initiate CyclinD_1_ transcription, and the increased expression of CyclinD_1_ can promote the activation of the pathway (Liu et al., 2021). Our results found that the expression of p-Tyr216-GSK-3β and p-β-catenin, the ratio of p-Tyr216-GSK-3β/GSK-3β and p-β-catenin/β-catenin in the brain of AD mice all increased significantly. However, these proteins were significantly downregulated by BLWE. These results indicated that the GSK-3β protein was activated, and the transduction of the Wnt signal was weakened in AD mice but can be reversed by BLWE treatment. Meanwhile, we also found that the expression of CyclinD_1_ protein was decreased significantly in the AD mice but increased after BLWE treatment. CyclinD_1_ a regulator of cyclin dependent kinases (CDKs), plays an important role in promoting cell proliferation and is a key molecule in regulating neurons from G_0_ to G_1_. Current study suggested that the therapeutic effect of BLWE in promoting neurogenesis in the treatment of AD may be achieved by initiating CyclinD_1_ transcription and then activating the Wnt/β-catenin signaling pathway.

In addition, we also focused on the astrocyte-mediated glutamate-glutamine cycle (GGC), which can support the metabolic demands and neurotransmitter transmission of neurons, promote the establishment of synaptic connections between neurons and form neural networks (Tani et al., 2014; Sidoryk-Wegrzynowicz and Struzynska, 2019). NAA and glutamine are precisely the key substances in GGC. Based on this, we speculate that the BLWE can promote neurogenesis, and treatment of AD may also be related to astrocytes, which will be the key point to our further research in the future.

## Conclusion

The present study evaluated the effects of BLWE in promoting neurogenesis in the treatment of AD and used metabolomics technology to screen different metabolites, and then accurately analyzed its mechanism. BLWE can promote the regeneration of neurons in the brain and establish synaptic connections. These effects may be achieved by activating the Wnt/β-catenin signaling pathway. These findings provide an experimental foundation for the application of BLWE in TCM clinical treatment of AD.

## Data Availability

The original contributions presented in the study are included in the article/Supplementary Material, further inquiries can be directed to the corresponding author.

## Glossary

AD: Alzheimer’s disease
Aβ: amyloid beta peptide
NMDA: N-methyl-D-aspartic aicd
AchE: acetylcholinesterase
NSCs: neural stem cells
CNS: central nervous system
APP/PS1: amyloid beta precursor protein/presenilin-1
Tg: transgenic
BL: *Broussonetia papyrifera* (L.) L'Hér. ex Vent. fruits
BLWE: *Broussonetia papyrifera* (L.) L'Hér. ex Vent. fruits water extract
GSK-3β: glycogen synthase kinase-3β
BrdU: 5-Bromo-2-deoxyuridine
MWM: Morris water maze
NeuN: neuronal nuclei
BSA: bovine serum albumin
SYN: synapsin
SYP: synaptophysin
PSD-95: postsynaptic density protein 95
ELISA: Enzyme-linked immunosorbent assay
SDS: sodium dodecyl sulfate
H&E: hematoxylin and eosin
PBS: phosphate buffered saline
TUNEL: terminal deoxynucleotidyl transferase dUTP nick end labeling
DAPI: 4,6-diamidino-2-phenylindole
NS: normal saline
HRP: horseradish peroxidase conjugated
ECL: electrochemiluminescence
LC: liquid chromatograph
MS: mass spectrometer
PVDF: polyvinylidene fluoride
HMDB: Human Metabolome Database
KEGG: Kyoto Encyclopedia of Genes and Genomes
PCA: principal component analysis
PLS-DA: partial least squares discriminant analysis
OPLS-DA: orthogonal partial least squares
SVZ: subventricular zone
SGZ: subgranular zone
TCM: Traditional Chinese medicine.
